# An Exploration of Rhythmic Grouping of Speech Sequences by French- and German-Learning Infants

**DOI:** 10.3389/fnhum.2016.00292

**Published:** 2016-06-14

**Authors:** Nawal Abboub, Natalie Boll-Avetisyan, Anjali Bhatara, Barbara Höhle, Thierry Nazzi

**Affiliations:** ^1^Laboratoire Psychologie de la Perception, Université Paris DescartesParis, France; ^2^Laboratoire Psychologie de la Perception, Centre National de la Recherche Scientifique (CNRS)Paris, France; ^3^Faculty of Human Sciences, Universität PotsdamPotsdam, Germany

**Keywords:** language acquisition, prosody, grouping, iambic-trochaic law, perceptual biases, french-learning infants, german-learning infants

## Abstract

Rhythm in music and speech can be characterized by a constellation of several acoustic cues. Individually, these cues have different effects on rhythmic perception: sequences of sounds alternating in duration are perceived as short-long pairs (weak-strong/iambic pattern), whereas sequences of sounds alternating in intensity or pitch are perceived as loud-soft, or high-low pairs (strong-weak/trochaic pattern). This perceptual bias—called the Iambic-Trochaic Law (ITL)–has been claimed to be an universal property of the auditory system applying in both the music and the language domains. Recent studies have shown that language experience can modulate the effects of the ITL on rhythmic perception of both speech and non-speech sequences in adults, and of non-speech sequences in 7.5-month-old infants. The goal of the present study was to explore whether language experience also modulates infants’ grouping of speech. To do so, we presented sequences of syllables to monolingual French- and German-learning 7.5-month-olds. Using the Headturn Preference Procedure (HPP), we examined whether they were able to perceive a rhythmic structure in sequences of syllables that alternated in duration, pitch, or intensity. Our findings show that both French- and German-learning infants perceived a rhythmic structure when it was cued by duration or pitch but not intensity. Our findings also show differences in how these infants use duration and pitch cues to group syllable sequences, suggesting that pitch cues were the easier ones to use. Moreover, performance did not differ across languages, failing to reveal early language effects on rhythmic perception. These results contribute to our understanding of the origin of rhythmic perception and perceptual mechanisms shared across music and speech, which may bootstrap language acquisition.

## Introduction

**Figure 1:**
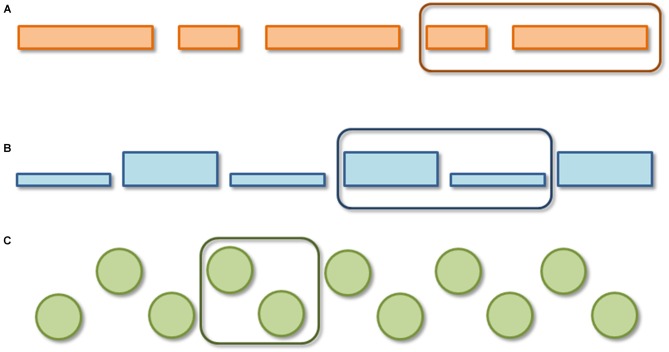
**Schematic of Iambic-Trochaic Law (ITL). (A)** Iambic duration grouping, **(B)** trochaic intensity grouping, **(C)** trochaic pitch grouping.

Perception of complex auditory patterns such as music or speech requires the auditory system to decompose or parse the acoustic signal into smaller units. One example is the segmentation of auditory input into chunks, known as perceptual grouping. An everyday example of this phenomenon is the “tick-tock” one hears when listening to a clock (Bolton, 1894) although the signal consists of re-occurrences of identical sounds. Importantly, such grouping is influenced by the duration, intensity, and pitch variation of the sounds. If the sequence alternates in duration (i.e., long-short-long-short), adult listeners group the sounds into iambic (weak-strong, final prominence) pairs (short-long, see Figure 1A). If a sequence alternates in intensity (i.e., loud-soft-loud-soft…) or pitch (i.e., high-low-high-low…) the opposite is true: adult listeners tend to group the sequence into chunks of a trochaic (strong-weak, initial prominence) pattern (i.e., loud-soft [see Figure 1B] or high-low [see Figure 1C]). This particular rhythmic grouping was initially demonstrated in the musical domain (Woodrow, 1909, 1911; Cooper and Meyer, 1960), and it was later extended to the linguistic domain and termed the *Iambic-Trochaic Law* (ITL; Hayes, 1995; Nespor et al., 2008). *Iambic* and *trochaic* are two possible stress patterns of words and/or prosodic constituents (such as phonological phrases), which constitute a language’s rhythmic structure. In many languages, words or phrases with initial prosodic prominence are often higher in intensity and/or in pitch on the first syllable, whereas words or phrases with final prominence are often longer in duration on the last syllable (Hayes, 1995; Nespor et al., 2008). This tendency affects the way individual listeners group sequences of sounds that alternate in one of these three acoustic cues. This effect has been demonstrated in adult listeners of numerous languages (French and German: Bhatara et al., 2013, 2015; Italian: Bion et al., 2011; French and English: Hay and Diehl, 2007). Based on the similarities in rhythmic structure between language and music as well as perceptual grouping preferences of speech and non-speech material, it has been proposed that the ITL may be a general auditory mechanism, governed by abstract, universal principles (Hayes, 1995; Iversen et al., 2008; Nespor et al., 2008; Yoshida et al., 2010; Bion et al., 2011).

Nevertheless, it has been hypothesized that a listener’s language environment might modulate the effects of the ITL, at least in part. Prosodic patterns as well as their acoustic correlates differ across languages. For example, in English and German, accentuation at the word level predominantly falls on the initial syllable in disyllabic words and is marked mostly by a contrast in intensity and/or pitch (trochaic pattern), whereas French has no lexical stress *per se* (Delattre, 1966; Dogil and Williams, 1999). Languages can also differ with respect to accent placement within the phonological phrase. For example, in English, phrases are iambic (i.e., emphasizing the last word in a phrase, e.g., to PARIS) whereas in Japanese, they are trochaic (i.e., emphasizing the first word in a phrase, e.g., TOKYO (Nespor and Vogel, 1986; Iversen et al., 2008; Nespor et al., 2008). German on the other hand, can follow either an iambic or a trochaic pattern. This phrasal distinction is caused by cross-linguistic differences in the syntactic parameter of head direction, which determines the possible order of heads and their complements in a given language. As the complement carries the prosodic prominence, head direction is associated with the position of the most prominent element within the phonological phrase. In head initial languages (e.g., French), the most prominent element is at the end of a phonological phrase, and in head final languages (e.g., Japanese), it is at the beginning (see Nespor et al., 2008). Note also that the acoustic realization of accentuation varies cross-linguistically. For example, in French, phrasal stress is mainly marked by phrase-final lengthening (hence, a durational iambic pattern); it is also marked by phrase-final pitch movement, corresponding to a pitch rise (iambic) if the phrase is sentence-internal, and a pitch fall if the phrase is sentence-final (trochaic; see Delattre, 1966; Jun and Fougeron, 2002).

This hypothesis of cross-linguistic modulation of the ITL has received support from recent adult studies showing that grouping preferences vary with language experience. Iversen et al. (2008) found that both English and Japanese listeners group sequences of complex tones varying in intensity as predicted by the ITL, that is, trochaically. However, English but not Japanese listeners grouped sequences varying in duration iambically, as predicted by the ITL; in fact, Japanese listeners did not display a consistent preference. Recent findings by Bhatara et al. (2013, 2015) comparing German and French listeners’ grouping of sequences of syllables provided further support for cross-linguistic modulation of rhythmic grouping. Both groups followed the ITL predictions for duration and intensity, but the German group showed more consistent performance than the French participants, reflecting a more stable bias. Additionally a pitch-based trochaic grouping preference was found only for the German listeners. The relative weakness in grouping preferences by French listeners when compared to German listeners can, however, be ameliorated when German is acquired as a foreign language in adulthood (Boll-Avetisyan et al., 2015b).

The authors of the above studies have argued that the cross-linguistic differences observed in their studies result from prosodic differences between the languages of the participants in either phrasal structure (for English/Japanese) or word stress (for German/French). If so, this raises the issue of how and when these cross-linguistic differences in the input give rise to cross-linguistic differences in perception (i.e., the nature and developmental trajectory of the mechanisms that generate rhythmic perception of speech/non-speech stimuli). Is the ITL present early in life or does it emerge later in development? How and when in infancy does it become modulated by language exposure? Does the use of all three cues (duration, intensity, pitch) follow the same developmental trajectory?

Recent developmental work has found early rhythmic grouping preferences as predicted by the ITL by 6 months of age. One of the first studies to explore this issue in English-learning infants presented them with complex non-speech tones alternating in duration or intensity and measured their detection of silences (Trainor and Adams, 2000). The results showed that 8-month-old infants perceived iambic groupings when duration was alternated, but they perceived no specific grouping when intensity was alternated. The authors interpret these results as an indication that grouping by duration and by intensity follow different developmental trajectories. The hypothesis of different trajectories for different cues is also supported by another study, which tested English-learning 6- and 9-month-olds on a grouping task using syllables instead of complex tones (Hay and Saffran, 2012). However, Hay and Saffran’s results showed the opposite pattern to those of Trainor and Adams (2000), with the 6-month-old infants grouping by intensity and not by duration and the 9-month-olds grouping by both intensity and duration. The discrepancy between these studies could be due to the materials used, but Hay and Saffran (2012) also tested the 9-month-olds on complex tones and found weaker but similar results to the syllables task; hence, it is unlikely that the difference is due strictly to the materials used. A more likely explanation of the difference between the studies is in the task. Hay and Saffran (2012) were not testing only rhythmic grouping; they also linked the prosodic cues to statistical cues (transitional probabilities), which may have changed the weight given by infants to the different cues. Together, these results nevertheless strongly suggest early use of intensity and duration for grouping, in accordance with the ITL, but also different developmental trajectories for rhythmic grouping based on these two cues.

A study by Bion et al. (2011) also showed variation in the developmental trajectory of the cues used for rhythmic grouping, this time between duration and pitch. Using the Headturn Preference Procedure (HPP), they familiarized two groups of 7.5-month-old Italian-learning infants with sequences of six syllables, presented repeatedly in the same order for 3 min, alternating either in duration (group 1) or in pitch (group 2). All infants were then tested on their perception of pairs of these syllables, presented without any acoustic/prosodic cues. Half of the test pairs of syllables had been presented with final prominence (short-long or low-high) in the familiarization phase, and the other half had been presented with initial prominence (long-short or high-low) in the familiarization phase. Results showed a preference for the initial prominence items in the pitch condition (interpreted as a familiarity effect), but failed to show a preference for either type of grouping in the duration condition. Similar results were obtained in a study on rats presented with sequences of complex tones; the rats showed evidence of rhythmic grouping by pitch but not by duration (de la Mora et al., 2013). A second study on rats further established that the emergence of duration-based grouping in rats is dependent on the nature of exposure: indeed, after being exposed to a regular duration-based pattern (pairs with either initial or final prominence), rats consistently grouped sequences varying in duration according to the pattern to which they had been exposed (Toro and Nespor, 2015). The authors interpreted these findings as possible evidence that grouping by pitch results from a universal perceptual bias shared across species, whereas grouping by duration would be more linked to auditory experience, and therefore would emerge later in human development after some exposure to language. In order to better understand the interplay of potential universal biases and the role of experience in rhythmic perception, and how these factors may depend on the type of acoustic cue, studies are needed that compare the use of pitch, intensity and duration cues in different languages, using the exact same procedures and stimuli.

Only two studies have looked at cross-linguistic differences on rhythmic grouping in infants, using the same procedures and materials for each linguistic group. The first study tested monolingual infants who were 5–6 months or 7–8 months old, learning either English or Japanese (Yoshida et al., 2010). The authors presented the infants with a 2-min familiarization sequence made up of complex tones alternating in duration. In the test phase, they measured the infants’ preference for pairs with final prominence (short-long) or initial prominence (long-short). Only the English-learning 7–8-month-olds showed any preference, and they preferred the pairs with an initial prominence. The Japanese-learning infants did not show a preference at either age. The authors suggest that the older English-learning infants were able to segment the sequence varying in duration into pairs with final prominence, and thus interpret the preference for pairs with initial prominence as a novelty preference. These results appear to extend the cross-linguistic differences found with Japanese- and English-listening adults to infancy (Iversen et al., 2008), and were taken as evidence that modulation of the ITL might be related to linguistic properties at the phrasal level. However, because there were no control sequences (without acoustic variation, or varying in another parameter) in the experiment, the possibility remains that this effect is due to English-learning infants’ bias for prominence-initial items, which has been demonstrated around this same age (Jusczyk et al., 1993), and not to sensitivity to the ITL *per se*.

The second cross-linguistic study used the same methods with tone stimuli as Yoshida et al. (2010) with a shorter familiarization (1 min 30 s), testing 9-month-old bilingual infants who were dominant in either Basque or Spanish, two languages that also differ in phrasal prosody with Basque having phrase-initial and Spanish having phrase-final stress (Molnar et al., 2014, 2016). Both duration- and intensity-varied sequences were tested. For intensity, both groups showed the same perceptual grouping and no linguistic modulation was found. However, for duration, cross-linguistic differences similar to those reported by Yoshida et al. (2010) were found: the Spanish-dominant infants had a preference for sequences with initial prominence, whereas the Basque-dominant infants had no significant preference, but showed a positive correlation between the amount of exposure to Basque and a preference for sequences with final prominence. On a more methodological issue, note that in both Yoshida et al. (2010) and Molnar et al. (2014), the preferences observed were attributed to novelty effects, assuming that they had grouped the familiarization string into short-long sequences (as expected by the ITL), and subsequently preferred novel long-short groupings over familiar short-long groupings. These new findings again suggest a link between modulation of the ITL and experience with the phrasal prosody of the native language.

In summary, previous research suggests that grouping preferences as predicted by the ITL emerge between 6 and 9 months and are partly modulated by linguistic experience. These studies suggest that a general auditory mechanism may be in place very early in infancy and may also be modulated by language exposure. However, the number of studies exploring grouping in infants is limited, and the results are mixed. Indeed, there is some evidence for the use of all of the three cues in at least one language and age group, but use is not found consistently. This raises questions regarding how this use is modulated across languages and development. Moreover, even though there appears to be a strong and early effect of language exposure, it remains poorly understood. So far, evidence from cross-linguistic studies has only revealed differences for sequences varying in duration. These results could suggest that grouping by duration is more dependent on language experience, whereas grouping by pitch or intensity are based on more general auditory mechanisms. Grouping by duration could be dependent on the prosodic properties of the language at the phrasal level (Iversen et al., 2008; Yoshida et al., 2010; Gervain and Werker, 2013; Molnar et al., 2014), although it has also been proposed that some modulation of the ITL might also stem from the word level (Bhatara et al., 2013, 2015). However, it is difficult to make generalizations like this based on the literature available thus far; the only two studies to test grouping in infants cross-linguistically (Yoshida et al., 2010; Molnar et al., 2014) differ in ages tested, length of familiarization, and acoustic cues tested. As mentioned earlier, no single study so far has tested all three of the cues cross-linguistically using the same methodology and using speech stimuli. Additionally, previous cross-linguistic studies testing infants have only compared languages differing in their *phrasal* stress (English/Japanese; Spanish/Basque). Here, we present a comparison of infants learning languages differing mostly in stress at the *word* level (French and German; see Bhatara et al., 2013), and we examine their perception of all three acoustic cues in syllable strings.

The present study is designed to answer the following questions: first, are all three acoustic cues (duration, intensity or pitch) used for grouping speech sequences at 7.5 months? If this were not the case, it would support differences in the trajectory of use for rhythmic grouping of the three cues. Second, is this grouping modulated by linguistic experience at this early age? Accordingly, we used methods similar to those of Bion et al. (2011), testing 7.5-month-old infants learning either French or German. If grouping by certain cues is modulated by linguistic experience at this age, we should see differences between these two groups that reflect the differences demonstrated in French and German adults (Bhatara et al., 2013, 2015). If, however, language has no effect on the ITL at this age, both groups should show similar patterns of grouping, that is, according to the ITL, trochaic grouping for the intensity and pitch conditions and iambic grouping for the duration condition.

In order to test these hypotheses, we used a familiarization-plus-test procedure using the HPP following Bion et al. (2011), and testing both French- and German-learning infants. As in that study, all infants were familiarized for 3 min with a continuous stream of the same six syllables, each infant being assigned to one of four conditions: three conditions each testing the use for grouping of a given cue (duration variation, intensity variation or pitch variation) and one control condition (no duration/intensity/pitch variation), as a baseline preference for the test items. Then, all infants were tested with two types of syllable pairs from these streams, presented without acoustic variation, and which had been presented with initial vs. final prominence in familiarization. In this specific HPP paradigm, rhythmic grouping of the familiarization sequence would be attested if infants demonstrated a differential response (measured in looking times) to the two types of stimuli in the test phase. In our case, based on Bion et al. (2011), we hypothesized that this difference in looking time would show that infants have memorized the stream as pairs of syllables that followed the rhythmic patterns predicted by the ITL (i.e., syllable pairs instantiating a short-long, loud-soft or high-low pattern in the stream).

What was less clear based on the literature was whether in the present study, infants would show a preference for the syllable pairs corresponding to the familiar (ITL-based) or the novel grouping. However, Hunter and Ames (1988) suggested that preferences in infants reflect the interaction among several factors, such as age, stimulus complexity and task difficulty. They proposed that infants typically display novelty preferences if the task is relatively easy (in the present case, if a cue is easy to use for grouping since age and task were constant across conditions) and familiarity preferences if the task is relatively complex. According to previous research investigating the ITL in infants reviewed earlier, using the same procedure (HPP; Yoshida et al., 2010; Bion et al., 2011; Molnar et al., 2014) and a procedure that does not rely on any novelty/familiarity interpretation (conditioned head turn; Trainor and Adams, 2000), the emergence of rhythmic grouping preference might differ across the three acoustic cues, meaning that some cues may be processed more easily than others. For this reason, we might expect that infants would have a familiarity preference for the cue(s) that are more complex for them to use for grouping and a novelty preference for less complex cue(s).

## Materials and Methods

### Participants

We tested a total of 205 monolingual full-term 7.5-month-old infants, learning either French in Paris, France, or German in Potsdam, Germany. There were four conditions (pitch, intensity, duration, and control) and two languages. Twenty infants were included in each condition/language combination (*n* = 160). We excluded 51 infants because of fussiness/tiredness (36 infants), having more than three insufficient looking times (<1500 ms; 4 infants), due to technical error (4 infants), being an outlier (i.e., with the difference between the mean orientation times of the two item types 2 SDs above or below the group mean; 5 infants) or other inability to finish the experiment (2 infants). See Table 1 for more details on the infants included in each condition. All parents gave informed consent before the experiment. The present experiment was approved in Paris by the ethics board “Conseil d’évaluation éthique pour les Recherches en Santé” (CERES) at the Université Paris Descartes and by the ethics board of the University of Potsdam.

**Table 1:** **Participant information of the four experimental conditions**.

	French				German			
Condition	Duration	Intensity	Pitch	Control	Duration	Intensity	Pitch	Control
Girls	12	10	9	8	10	12	10	10
Boys	8	10	11	12	10	8	10	10
Mean age in months (SD)	7.5 (0.3)	7.4 (0.3)	7.5 (0.3)	7.5 (0.2)	7.6 (0.3)	7.4 (0.3)	7.2 (0.2)	7.5 (0.4)
Range (months)	7.1–8.0	7.0–7.9	7.1–8.0	7.1–7.8	7.1–8.0	7.0–8.0	7.1–7.8	7.0–8.3

### Stimuli

As in Bion et al. (2011), the stimuli were sequences of six syllables created by combining six vowels (/a:, e:, i:, o:, u:, y:/) with six consonants (/f, n, g, p, r, z/). These were selected for two reasons: first, they are phonemes that exist in both French and German, even if their realizations differ slightly. The result of this was that the segmental variability was the same for the two language groups. Second, the vowels and consonants vary phonologically (vowel roundness, height, and place, and consonant voicing, manner, and place). These syllables were concatenated into a stream in such a way that it contained no disyllabic words in either language: /na: zu: gi: pe: fy: ro:/. Syllables were separated by a pause of 100 ms. For the four different conditions, we created sequences in which syllables alternated in either duration, intensity, pitch, or nothing (control condition), see Supplementary Figure S1 in Supplementary Material. The sequences were synthesized using two female voices in MBROLA (Dutoit et al., 1996), one French (fr4) and one German (de5)^1^.

In the familiarization phase, the six-syllabic sequence was repeated 66 times, in order to last about 3 min. The acoustic variation was added to the sequence using a combination of MBROLA and PRAAT (Boersma and Weenink, 2010). The duration manipulation was applied to the vowels and pitch and intensity at the whole syllable. The values of duration, intensity and pitch variation were chosen in order to stay close to the values naturally present in these two languages (e.g., Bhatara et al., 2013) while also attempting to replicate Bion et al. (2011) as closely as possible (see Table 2 for a summary of the values). Note that the baseline and control values for the intensity (70 dB) and duration (360 ms) conditions were also the means of the variation condition. However for the pitch condition, because the baseline would have been too high and sounded unnatural if we had used the mean of the variation we chose (200–420 Hz, so 310 Hz), we decided to use 200 Hz as baseline.

**Table 2:** **Acoustic variation values for each condition**.

	Condition	Pitch (Hz)	Intensity (dB)	Duration (ms)
Familiarization	Duration	200	70	260–460
	Intensity	200	66–74	360
	Pitch	200–420	70	360
	Control	200	70	360
Test	(All Conditions)	200	70	360

Several previous studies on rhythmic grouping found a strong influence of the onset of the sequence on perceived grouping. The first two sounds heard tend to serve as an anchor point (Woodrow, 1909; Trainor and Adams, 2000; Hay and Diehl, 2007). For this reason, we created “ramps” to mask the onset of each sequence in two ways. The first aspect of the sequence onset mask was inspired by Hay and Diehl (2007) and used in the same way as Bhatara et al. (2013): we added white noise masking over the first four repetitions of the sequence (10 s), decreasing in intensity as the sequence itself increased in intensity from silence, with both the increase and the decrease following a raised cosine function. The second aspect of the sequence onset masking ramp was inspired by Bion et al. (2011), who inserted a gradual increase of the rhythmic cue, starting on the third syllable. For example, in the duration condition, the first two syllables had equal duration (260 ms) and starting with the third syllable, every odd syllable increased by 20 ms. Thus, the third syllable was 280 ms long, the fourth 260 ms, the fifth 300 ms, the sixth 260 ms, and so on until the maximum of 460 ms was reached, so that every odd syllable was the longer one. In our study, to counterbalance the start of the increasing variation, half of the increases started on the fourth rather than the third syllable, so that every even syllable was the longer one. This resulted in two different ramp types. Similar manipulations were performed in the pitch and intensity conditions. The pitch condition started at 200 Hz and increased by 20 Hz every other syllable until it reached 420 Hz, and the intensity condition started at 66 dB and increased by 1 dB every other syllable until it reached 74 dB.

The items used at test corresponded to the six disyllables that had occurred during the familiarization (/na:zu:/, /zu:gi:/, /gi:pe:/, /pe:fy:/, /fy:ro:/, /ro:na:/). Crucially, during the test phase, both syllables of a test item were equal in pitch, duration, and intensity; hence, preferences observed could not depend on the acoustic/prosodic properties of the syllables at test. Half of these disyllables had been presented with final prominence (i.e., short-long, low-high, or soft-loud, depending on the condition) and the other half with initial prominence (i.e., long-short, high-low, or loud-soft) in the familiarization. Six sound files were prepared, each containing one of the six test items repeated 16 times, lasting 14.5 s. Each of the six sound files was presented twice, leading to a test phase of 12 trials, and a different random order of presentation of the trials was used for each participant. The test items were synthesized with the same MBROLA voice the participants had heard during the familiarization phase.

### Procedure Apparatus and Design

We used the HPP (Kemler Nelson et al., 1995) for this study. The infants were seated comfortably on their parents’ lap in a soundproof booth. A green light was directly in front of the infant. There was a red light on each side of the room, at the same height as the green light. Speakers were hidden behind the wall underneath the red lights. The experimenter sat outside the booth and observed the infants using a camera under the green light. The experimenter controlled the stimulus presentation and the blinking of the lights according to the head movements of the infant by pressing three buttons on a button-box (one for each light, see further details below). Both the experimenter and the parent wore headphones playing music that masked the stimuli.

The experiment began with the familiarization phase. The infant heard one of the four-familiarization sequences (duration, intensity, pitch, or control) from both speakers simultaneously via an amplifier. Half of the infants heard a sequence of familiarization with the acoustic variation starting on the third and half heard variation starting on the fourth syllable. Additionally, half of the infants in each condition and in each language heard the German voice, and half heard the French voice. During this phase, the lights blinked according to where the infant looked, but the sound was not presented contingently on the infant’s behavior. In the test phase, which was the same across all four conditions, all infants heard 12 trials: three disyllables that had had initial prominence and three that had had final prominence during familiarization, each presented twice (in order to simplify the item labeling, we use the terms “initial prominence” and “final prominence” even though all disyllables were free of acoustic variation in the test phase). The trials were presented in a different random order for each participant. The side of the loudspeaker from which the stimuli were presented was randomly varied from trial to trial, with the constraint that 1/2 of the trials of each kind were presented on each side.

Each trial began with the green light blinking in order to center the infant’s gaze. After the infant looked at the green light, the experimenter pressed a button to make the red light on one of the side panels start blinking. When the infant looked at the red light, the sound and the trial began. The side of the loudspeaker from which the stimuli were presented was randomly varied from trial to trial. If the infant turned away from the light for more than 2 s while the sound was playing, both the blinking and the sound stopped, and the green light began blinking again. If the infant looked away and then back again within the period of 2 s, the sound continued to play. However, this time of looking away from the light was subtracted from the total looking time for that trial. Information about the duration of the head turn was stored on the computer.

## Results

Looking times for the prominence-initial and prominence-final items were averaged across each participant. Note that test items in the control condition cannot be referred to as having initial or final prominence because the syllables were all at baseline pitch, intensity and duration. However, to explore whether the onset of the sequences served as anchor points for grouping (as in Woodrow, 1909; Trainor and Adams, 2000; Hay and Diehl, 2007), we decided in the control condition to label the pairs that would be segmented by using the first syllable to initiate the grouping process (1–2, 3–4, and 5–6) as “prominence-initial,” and the other pairs, corresponding to starting the grouping from the second syllable (2–3, 4–5, and 6–1), as “prominence-final.”

To determine whether infants process the two “types” of pairs (prominence-initial vs. final) as predicted by the ITL for the duration, intensity and pitch duration but not for the control condition, the mean looking times for the two types were averaged across infants, for the first vs. last three trials of each type (in order to be able to explore block effects). We performed repeated-measures ANOVAs on these mean looking times with the within-subjects factor of block (first vs. last three trials of each item type) and the between-subjects factors of condition (duration, intensity, pitch, or control). There was a strong effect of block, *F*_(1,156)_ = 81.82, *p* < 0.00001, $\eta_{p}^{2}$ = 0.34 and an effect of condition *F*_(3,156)_ = 2.70, *p* = 0.048, $\eta_{p}^{2}$ = 0.049. No other effects or interactions were significant.

The lack of an effect or interactions involving item type (prominence-initial and prominence-final items) indicates a failure to show grouping. However, the results show that infants’ looking times significantly decrease throughout the experiment (from a mean of 8.67 s in the first trial to 6.24 s in the last trial), independently of the condition. For this reason, we decided to run a second ANOVA restricted to the first block of each item type, since more transient effects might be observed in the earlier part of the test phase (see further discussion of this point in the “General Discussion” Section).

**Figure 2:**
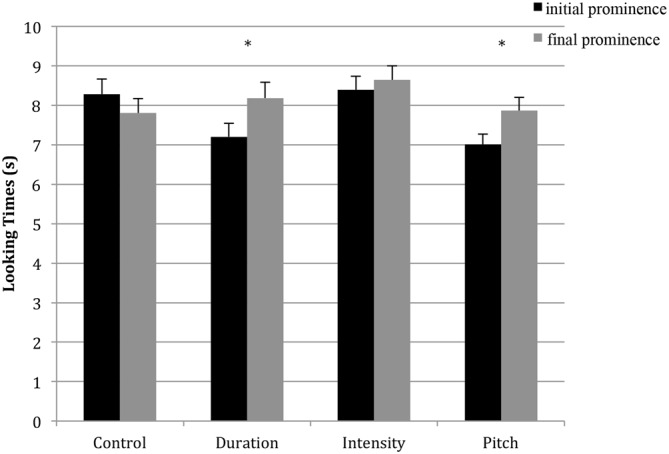
Looking time for initial prominence and final prominence items for each condition, *p < 0.05.

Results restricted to the first block are shown in Figure 2. We performed a repeated-measures ANOVA on these mean looking times with the within-subjects factor of item type (prominence-initial or -final) and the between-subjects factors of native language (French or German) and condition (duration, intensity, pitch, or control). There was a significant effect of item type, *F*_(1,152)_ = 4.76, *p* = 0.03, $\eta_{p}^{2}$ = 0.03, as well as a significant interaction between item type and condition, *F*_(3,152)_ = 3.29, *p* = 0.022, $\eta_{p}^{2}$ = 0.06. These results show that infants differentiate between disyllables that had initial vs. final prominence during familiarization, and that the way they differentiate them changes depending on the condition. There were no other significant effects.

Next, in order to more closely examine the interaction between item type and condition, we analyzed each condition separately. For the control condition, a simple *t*-test for item type was conducted. For the other three conditions, repeated-measures ANOVAs were conducted with the factors of item type and ramp type (whether the variation started on the third or fourth syllable).

### Control Condition

There was no effect of item type, *t*_(39)_ = 1.41 *p* = 0.16. Infants looked equivalently at the “prominence-initial” items (*M* = 8.28 s) and the “prominence-final” items (*M* = 7.81 s), suggesting that during the test phase, the infants had no particular preference for specific pairs of syllables following the familiarization sequence that was neutral in terms of promoting ITL-based grouping.

### Duration Condition

There was a significant main effect of item type, *F*_(1,38)_ = 9.88, *p* = 0.003, $\eta_{p}^{2}$ = 0.21, with infants looking longer for prominence-final items (*M* = 8.18 s) than for prominence-initial items (*M* = 7.21 s). There was also a significant interaction between ramp type and item type, *F*_(1,38)_ = 6.34, *p* = 0.016, $\eta_{p}^{2}$ = 0.14. No other effects or interactions were significant.

To better understand the interaction between ramp and item type, we examined the effect of item type on each ramp type. It appears that the infants looked longer for prominence-final items when the ramp started on the fourth syllable (*M_final_* = 8.97 s vs. *M_initial_* = 7.21 s), *t*_(19)_ = 3.83, *p* < 0.001, whereas there was no looking time difference if the ramp started on the third syllable (*M_initial_* = 7.20 s vs. *M_final_* = 7.39 s), *t*_(19)_ = −0.46, *p* = 0.65.

### Intensity Condition

There were no significant effects or interactions for intensity-varied sequences, indicating that infants did not show any grouping preference.

### Pitch Condition

For the pitch-varied sequences, there was a significant effect of item type, *F*_(1, 38)_ = 4.78, *p* = 0.035, $\eta_{p}^{2}$ = 0.11. Infants looked longer for prominence-final items (*M* = 7.87 s) than for prominence-initial items (*M* = 7.01 s). There were no significant effects of ramp type and no significant interactions.

## Discussion

In this set of studies, we have examined French- and German-learning infants’ rhythmic grouping of linguistic sequences according to the ITL. Our first research question was whether German- or French-learning infants by 7.5 months of age would group linguistic sequences according to the ITL: that is, prominence-initial for intensity and pitch and prominence-final for duration. Second, we wanted to evaluate whether this ITL-based grouping is already modulated by native language experience at that age.

In both the duration and the pitch conditions, both German- and French-learning infants at test looked longer at the items that had been prominence-final in the familiarization. Given that there was no preference for the test items in the control condition, this shows that the preferences observed here are induced by the specific properties of the two familiarization conditions. Hence, it appears that 7.5-month-old infants learning either language can make use of duration and pitch cues to segment syllable sequences. For the intensity condition, there was no significant effect. Below, we discuss the results of the separate conditions followed by a comparison of the results of the separate conditions in an integrative discussion.

### Control

We did not find any evidence for a default grouping in the 7.5-month-olds in our study when no acoustic variation was present in the familiarization sequence. Previous studies with adults have found a default trochaic grouping of sequences without any acoustic variation of the relevant cues in native speakers of English (Rice, 1992; Hay and Diehl, 2007) and of German but not of French (Bhatara et al., 2013), unless native speakers of French had second language knowledge of German (Boll-Avetisyan et al., 2015b). These results suggest that under the present experimental conditions (i.e., with segmental variability and without any prosodic cues), infants at 7.5 months of age do not segment the familiarization sequence into bisyllabic chunks. The lack of a preference for any of the syllable pairs presented during the test phase is important to note because it indicates that any preferences found in the other familiarization conditions do not result from intrinsic preferences for some of these pairs (since test pairs were identical across all four conditions) but instead reflect preferences induced by rhythmic grouping. Moreover, this null-result may suggest that without any acoustic cues, infants do not group the sequence at that age, and that the default trochaic segmentation bias found in English and German adults emerges later in development.

### Duration

For the duration-varied condition, we found a preference for prominence-final items over prominence-initial items in the test phase, establishing that the infants used the duration variation in the familiarization phase to group syllables into pairs. This grouping then induced a preference at test, a conclusion based on the fact that no preference was found for the same test items following familiarization in the control condition (see details above). Additionally, if we assume that the infants grouped syllables iambically during the familiarization as predicted by the ITL for duration-varied sequences, then in the test phase, they appear to listen to the syllable pairs that were “familiar” given the familiarization phase.

This familiarity effect is in the opposite direction from results of previous studies, which had shown that 7–8-month-old Canadian English-learning infants (Yoshida et al., 2010) and 9-month-old bilingual Spanish/Basque-learning infants (Molnar et al., 2014) have preferences for prominence-initial items at test. Both sets of authors interpreted these results as indicative of a novelty preference, according to the model proposed by Hunter and Ames (1988). Because familiarity preferences may arise instead of novelty preferences when infants are younger or find the tasks more difficult (Hunter and Ames, 1988), we hypothesize that the present familiarity preference is a consequence of the fact that either the task or material used in the present study might be more difficult than that of Yoshida et al. (2010) or Molnar et al. (2014). The present task included a long familiarization sequence (3 min, as in Bion et al., 2011) relative to previous studies (2 min in Yoshida et al., 2010 and 90 s in Molnar et al., 2014), which should have led, if anything, to an even stronger novelty effect. However, our task might have been more difficult because infants had to memorize the syllables during familiarization in order to show a preference in the subsequent test phase. In contrast, in Yoshida et al. (2010) and Molnar et al. (2014), the test phase (with stimuli including acoustic cues for prominence) tested for a relative preference for trochaic over iambic stress patterns, and this task did not require memorizing and recognizing the familiarization stimuli. Furthermore, the preference for the trochaic pattern found in these studies could have resulted, at least for English, from the emergence of infants’ preference for trochaic words around that age (Jusczyk et al., 1993). Moreover, regarding the material, the stimuli in both previous studies were sequences of tones, whereas we presented infants with sequences of syllables, that is, with much more acoustic variation created by the segments forming the syllables. Since more complex stimuli have been shown to be more difficult to process in ITL-related tasks for both French and German adults (Bhatara et al., 2015), it would be reasonable to assume that the same would be true for infants. Hence, both our task and stimuli might be more difficult than in Yoshida et al. (2010) and Molnar et al. (2014), which might explain why we found a familiarity rather than a novelty preference for the ITL-based short-long pattern.

Moreover, our findings also appear to differ from those of Bion et al. (2011), which did not show sensitivity to duration for grouping in Italian-learning infants at the same age, even though our method was closely replicating the method they used. However, there was one important difference between the two studies; in Bion et al. (2011), the prominent syllables were always the odd syllables of the syllable sequence, whereas in the current study, the position of prominent syllables was counterbalanced (on odd syllables for half of the infants, on even syllables for the other). This counterbalancing effect turned out to have a marked impact on performance in our infants (in spite of the onset of the sequence being masked by fading-out white noise and fading-in intensity in addition to the step-wise increase of the alternation), replicating similar effects in both adults and infants (Trainor and Adams, 2000; Hay and Diehl, 2007). Indeed, our findings show that when the duration variation was on odd syllables (as in Bion et al., 2011), French- and German-learning infants also failed to show evidence of having grouped the syllables of the familiarization sequence. However, when the duration variation was on even syllables, infants succeeded.

How can we explain this ramp/positional effect? One possibility is that infants also use a default grouping mechanism that extracts disyllables aligned to the onset of the sequence they are presented with. When this default mechanism is aligned with ITL-based grouping (extracting short-long pairs) as is the case when stress is on even syllables, then both mechanisms would converge in their grouping results. Note however that this default mechanism is probably not very powerful, as attested by the lack of grouping effects in the control condition (where it should have given odd-even syllable sequences), and in the pitch and intensity conditions (where it should have induced larger effects in the sequences with variation on the odd syllables, which would have aligned with prominence-initial ITL-based grouping). If this interpretation is correct, then it is possible that Bion et al. (2011) would have found a grouping preference for duration if they had presented the ramp starting on the fourth syllable. Note that this ramp effect is another indication that, at least in this task, duration may be a grouping cue difficult to use at 7.5 months of age (see more on this point regarding the differing pattern of results in the pitch condition).

Hence, our study adds to the existing literature on infants with different native languages (English, bilingual Spanish/Basque [Spanish-dominant], French, and German), which have shown that infants group sound sequences by duration. This literature further shows that some infants, namely Japanese and Spanish/Basque [Basque-dominant]-exposed infants, do not show grouping preferences based on duration. These results suggest that several factors influence rhythmic grouping development, including both sequence structure and native language. In our study, it may be that the familiarity preference shows that our French- and German-learning 7.5-month-olds still found it difficult to use the duration cue present in our stimuli (as it is found only in one ramp condition).

### Pitch

In the pitch condition, we found that infants at test preferred listening to items that had had final prominence during the familiarization phase, establishing that they used pitch to group the syllables in the familiarization sequence. Considering prior experiments with adults and infants (Bion et al., 2011; Bhatara et al., 2013; Gervain and Werker, 2013) showing ITL-based trochaic grouping of pitch-varied sequences, these results would indicate a novelty preference. Given the fact that we found a familiarity effect in the duration condition, and given the Hunter and Ames (1988) model, this novelty preference would suggest that it was easier for our French- and German-learning 7.5-month-olds to group the syllables based on pitch than on duration variation. This interpretation is independently supported by the fact that the effects in the pitch condition were not modulated by the ramp used, contrary to what was found in the duration condition, suggesting more stable use of pitch than duration to group in our experiments. In addition, recall that Bion et al. (2011) found a familiarity preference in their pitch-varied stimuli among Italian infants, that is, a preference for prominence-initial items. Hence, it also appears that it was easier for the French- and German-learning infants than it had been for the Italian infants to use pitch. Since both studies used very similar materials (synthesized non-word syllable sequences) and methods, it is possible that this difference relates to the infants’ native languages, but other cross-linguistic studies on grouping by pitch are needed to further explore this difference.

### Intensity

In the intensity condition, there were no significant effects in the analysis. To our knowledge, all previous studies who tested grouping by intensity used tones (Trainor and Adams, 2000; Molnar et al., 2014) or presented an artificial speech stream including cues from transitional probabilities between syllables along with the acoustic cues (Hay and Saffran, 2012). The present study used speech stimuli, and there were no cues from transitional probabilities between syllables. Hence, the reason that previous studies (Trainor and Adams, 2000; Molnar et al., 2014) but not the present study found effects could be due to methodological differences, either in complexity of material or in lack of additional cues. Another possible explanation for the present lack of grouping by intensity is that intensity alone is not by itself a relevant cue for infants’ processing of rhythm in language. Specifically, it is difficult to tease apart the effect of pitch and the effect of intensity given the fact that sounds with a higher pitch tend to be perceived with higher intensity (Fry, 1955; van Heuven and Menert, 1996; Mattys and Samuel, 2000). It has been shown that 7.5-month-old infants are sensitive to pitch variations in a lexical recognition task but ignore intensity variations (Singh et al., 2008). Taken together, these results suggest that intensity can only be used for grouping in combination with other cues (e.g., transitional probabilities and other prosodic cues). This possibility will have to be tested in future research.

### General Discussion

The present findings are in part consistent with the predictions of the ITL. Nevertheless, our results show that there are differences in the way rhythmic cues are used for rhythmic grouping. Whereas we did not find an effect in the control or intensity conditions, we found a *familiarity* effect in the duration condition that was additionally modulated by the position of the ramp and a *novelty* effect in the pitch condition that was not affected by the position of the ramp. It is possible that such differences reflect different perceptual mechanisms being at play. Based on previous studies (Bion et al., 2011; de la Mora et al., 2013), one possible interpretation of these results is that a more stable and consistent grouping for pitch reflects a general, universal auditory processing mechanism, although—because pitch in developmental populations was only previously tested in Bion et al. (2011)—this hypothesis it is still under debate. In contrast, grouping by duration and intensity may be more context-dependent and/or more affected by language experience. This interpretation is supported by the results of previous studies (including data on rats: de la Mora et al., 2013; Toro and Nespor, 2015). First, the finding that the use of duration as a cue for grouping depends on language background has been found in several studies in both adults (Iversen et al., 2008; Bhatara et al., 2013, 2015; Crowhurst and Teodocio Olivares, 2014; Boll-Avetisyan et al., 2015b) and infants (Yoshida et al., 2010; Molnar et al., 2014). It is therefore possible that, at least at the beginning of life, before full mastery of an infant’s native language, the specific pattern heard in their auditory environment affects rhythmic grouping based on duration but not pitch. However, our study is the first to test all three cues at the same time, and the emerging pattern of results remains difficult to fully interpret. Future studies comparing all three acoustic cues are needed, using different familiarization times, different levels of prosodic variability (in the present study, we only used one value per cue, making it difficult to evaluate the relative weight of each cue), and different types of materials (in particular, comparing linguistic and non-linguistic stimuli, see below).

Moreover, in the present study the effect of cue-based grouping was present only in the first part of the test phase. Indeed, in our first analysis analyzing all the trials, we found no significant effect or interaction involving test item type, but a significant decrease in looking times over the course of the test phase. This decrease in looking times is not surprising for two reasons: first, our familiarization phase was quite long when compared to other grouping experiments. Hence, it is not unexpected that the infants’ attention cannot be maintained over the course of this long experimental session. Second, it is not unexpected that memory for grouped items is freshest immediately after the familiarization phase. Adult segmentation studies have also reported that preferences for the “segmented” items are most pronounced in the initial portion of the test phase (e.g., Boll-Avetisyan and Kager, 2014). Hence, it is reasonable that infants’ preferences for, for example, prominence-final items in the duration condition are stronger in the initial portion of the test phase, immediately after they have been exposed to the stream. Further studies will have to take into account this effect in the design of this type of task.

Another interesting aspect of our results is the lack of cross-linguistic differences. We found the same listening biases for all infants independently of whether they are acquiring French or German. Until now, rhythmic grouping based on duration has generally been shown to be modulated by language exposure. Therefore, our results might be surprising at first, but they can be interpreted based on prosodic properties of the languages we tested, in particular in terms of their acoustic cues and position of lexical stress. Remember that Yoshida et al. (2010) as well as Molnar et al. (2014) compared languages with prominence at the end of the phonological phrase (English, Spanish) to languages with prominence at the beginning (Japanese, Basque). In contrast, French and German are less different on this level than the languages used in these previous studies, with phrases being prominence-final in French and predominantly prominence-final in German (even if both prominence-final and prominence-initial phrases are allowed in German). In contrast, these two languages greatly differ in terms of prosody at the lexical level. Because of the overall similarity with respect to the position of phrasal prominence, French- and German-learning infants’ perception of rhythmic groups might still be similar at 7.5 months of age. Hence, a comparison of infants learning two less similar languages would be more likely to demonstrate crosslinguistic differences, as Yoshida et al. (2010) and Molnar et al. (2014) have shown in Japanese/English and Basque/Spanish comparisons. Although cross-linguistic differences in ITL-based grouping were found between French and German adults (Bhatara et al., 2013, 2015), it appears that these cross-linguistic differences might be set into place later in development for languages that mostly differ in prosody at the lexical rather than the phrasal level.

A corollary to this discussion on developmental changes in the weighting of rhythmic cues has been observed in infants’ processing of prosodic boundaries in sentences. Studies by Seidl (2007) and Seidl and Cristià (2008) have shown that 4- and 6-month-old English-learning infants perceived acoustic cues of prosodic phrase boundaries differently (Seidl and Cristià, 2008). At 6 months, a pitch change but no lengthening or pause was necessary to perceive the boundary whereas at 4 months, infants needed a combination of all three of these boundary cues. Furthermore, this weighting and its developmental trajectory seems to vary cross-linguistically, as 6-month-old Dutch- and German-learning infants have been found to rely more heavily on the pause than their English-learning age mates, and German-learning infants are able to detect a phrase boundary that is solely marked by pitch and lengthening at the age of 8 but not 6 months (Seidl, 2007; Johnson and Seidl, 2008; Wellmann et al., 2012). These developmental changes might be linked to the nature of linguistic exposure, but also, in some part, might be related to infant directed speech (IDS) acoustic cues. IDS relative to Adult Directed Speech (ADS) has a generally slower tempo, higher average pitch, and exaggerated intonation contours (Fernald and Simon, 1984). These cues can differ in terms of strength according to the age of the infant (Kitamura and Burnham, 2003) and can also influence developmental changes in listening preference (Panneton et al., 2006) and might impact rhythmic grouping. Based on this reasoning, it is possible that, at the beginning of language acquisition, infants are relying on the ITL and general auditory perception, and that language-specific strategies will emerge later depending of the nature of the language exposure. Hence, the present study provides further evidence for a link between basic auditory processing and speech processing in language acquisition.

These results more broadly contribute to the common auditory skills account of speech, music and sound processing (Patel, 2003; Asaridou and McQueen, 2013) and particularly rhythmic processing for linguistic and non-linguistic sounds (Hay and Diehl, 2007; François et al., 2013; Bhatara et al., 2015; Boll-Avetisyan et al., 2015b). Rhythmicity characterizes many physiological processes and is widely acknowledged to be an important feature of both speech and music. Here for the first time we show early sensitivities for grouping linguistic sequences varying in pitch and duration in French- and German-learning infants, finding results similar to those from previous studies that presented infants with non-linguistic tones. These findings suggest that speech and non-speech sounds are processed by common mechanisms, although further support to this claim would be provided by studies directly comparing the processing of linguistic and non-linguistic stimuli. Although this has not been done for early rhythmic grouping (with the exception of Hay and Saffran, 2012), several studies have shown links between musical and linguistic perception at different other levels. Behavioral research has highlighted similarities in terms of structural processing for musical and linguistic sequences in adults and infants (Krumhansl and Jusczyk, 1990; Fedorenko et al., 2009). Neuroimaging studies have also found common networks for structural processing of speech and music (Patel, 2003; Abrams et al., 2011; Fedorenko and Thompson-Schill, 2014). There is also evidence that musical experience can influence rhythmic grouping of both speech and non-speech sequences by duration and intensity (Bhatara et al., 2015; Boll-Avetisyan et al., 2015b).

In summary, the present study has shown, for the first time using linguistic stimuli, grouping preferences based on pitch and duration variation in 7.5-month-old French- and German-learning infants. These results suggest that these perceptual grouping mechanisms are in place early in development. In our experiments, infants were tested in a speech segmentation task, in which they succeeded using both pitch and duration cues for segmentation. Because pitch and duration are relatively reliable cues to word- and phrase boundaries in natural speech, it is possible that infants use the same mechanisms that they relied on in the present task to segment words and phrases from natural speech. Moreover, we found no cross-linguistic differences for these cues, in contrast with previous studies examining similar questions in French and German adults (Bhatara et al., 2013, 2015). This suggests that, at 7.5 months, language experience might have begun to shape these mechanisms (as directly found in Yoshida et al., 2010; and Molnar et al., 2014; and indirectly by comparing our findings with those of Bion et al., 2011), but that some cross-linguistic differences might take longer to emerge (as in the present case of French and German). Moreover, our study contributes to the view that rhythmic grouping preference for speech may emerge from general perceptual biases. Recent studies have shown that groupings similar to those formalized by the ITL may even be found across species (de la Mora et al., 2013; Toro and Nespor, 2015) and across modalities (Peña et al., 2011). These studies reinforce the idea that these auditory biases would have evolutionary bases and that these biases that infants might use to segment the speech stream into lexical and/or phrasal units would rely on auditory mechanisms not specific to language processing, which might be present very early on in development. To get an even more precise view of the development of these abilities, future studies will have to further explore the roles of pitch, duration and intensity in speech and their emergence as grouping cues, testing infants with different native languages at different ages.

## Author Contributions

All authors participated in designing the experiments, interpreting the results and writing the article. NA, NB-A and AB prepared the stimuli, ran the experiments and analyzed the data.

## Conflict of Interest Statement

The authors declare that the research was conducted in the absence of any commercial or financial relationships that could be construed as a potential conflict of interest. The reviewer MB and handling Editor declared their shared affiliation, and the handling Editor states that the process nevertheless met the standards of a fair and objective review.

## Footnotes

^1^Prior experiments with duration, intensity or pitch cues were run using eight syllables rather than six syllables and without pauses between the syllables, making the task more difficult. These experiments revealed no evidence of ITL-based responses in 7 month-old French- or German-learning infants (Boll-Avetisyan et al., 2015a,b).

## Supplementary Material

Figure S1 **Segment from the middle of the familiarization stream with the three test conditions: (A) Intensity condition, (B) Pitch condition, and (C) Duration condition**.
