# Supplementary material for: An Exploration of Rhythmic Grouping of Speech Sequences by French- and German-Learning Infants
Source: Front Hum Neurosci. 2016 Jun 14;10:292. doi: 10.3389/fnhum.2016.00292 (PMC4906042; doi:10.3389/fnhum.2016.00292)
Supplement: Figure S1 — Segment from the middle of the familiarization stream with the three test conditions: (A) Intensity condition, (B) Pitch condition, and (C) Duration condition. [file Image_1.pdf]

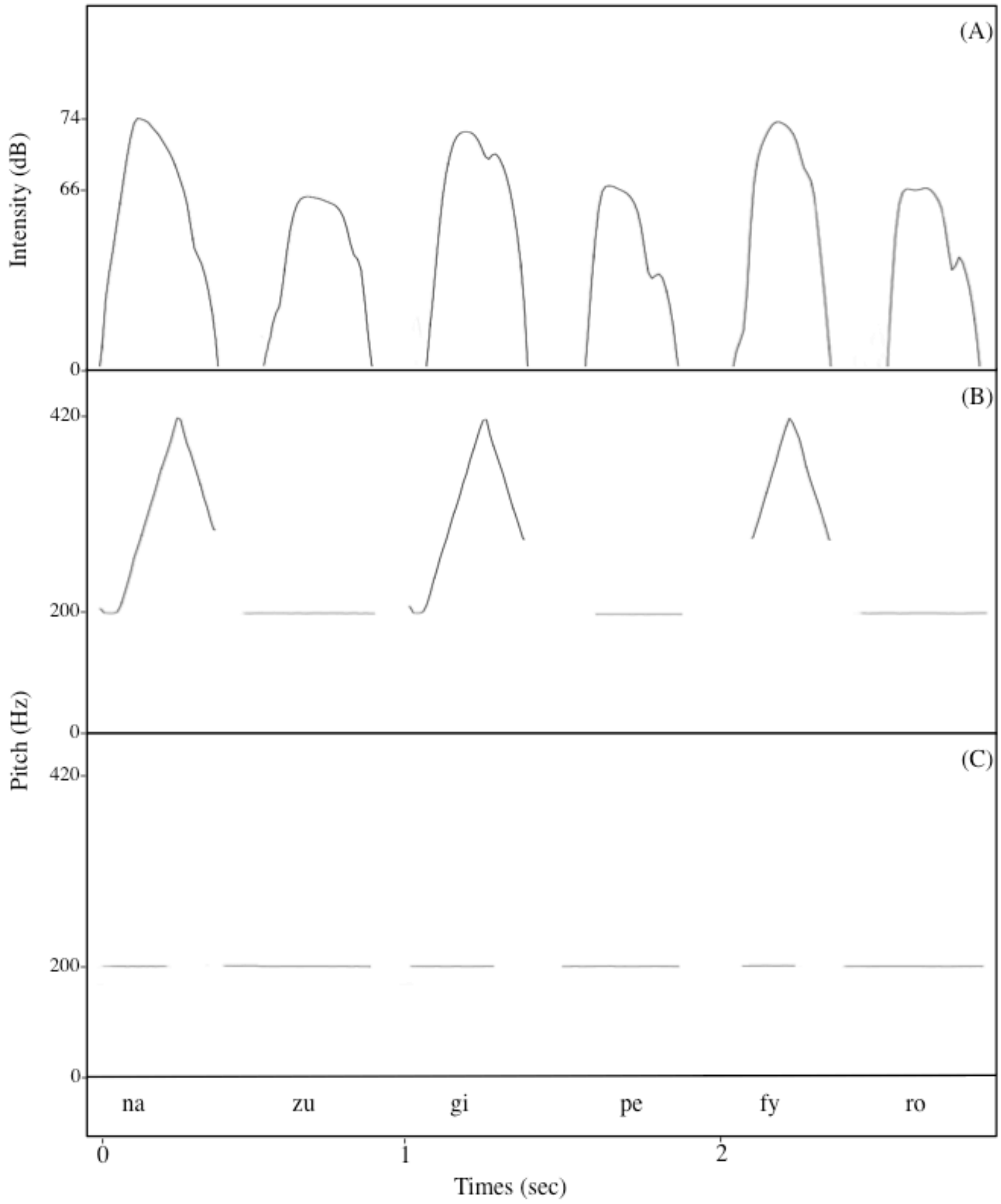

Figure S1: Segment from the middle of the familiarization stream with the three test conditions : (A): Intensity condition, (B): Pitch condition, (C): Duration condition.
